# Retrospective Study on the Risk of Nerve Injury After Distal Biceps Tendon Repair Using Cortical Button

**DOI:** 10.7759/cureus.43512

**Published:** 2023-08-15

**Authors:** Mohammed Tayyem, Omar Naji, Adesina Adetokunbo, Humam Jundi, Aniruddha Pendse

**Affiliations:** 1 Trauma and Orthopaedics, Buckinghamshire Healthcare National Health Service (NHS) Trust, Aylesbury, GBR

**Keywords:** retrospective study, superficial radial nerve, lateral cutaneous nerve, posterior interosseous nerve, nerve injury, cortical buttons, distal biceps tendon

## Abstract

Background: Distal biceps tendon ruptures are relatively rare injuries that typically require surgical intervention to restore flexion and supination strength. Concerns have been raised regarding the risk of nerve injuries, particularly the posterior interosseous nerve (PIN), associated with the use of cortical buttons in distal biceps repair. This study aimed to estimate the incidence of PIN injury as well as injuries to the lateral cutaneous nerve of the forearm and superficial branch of the radial nerve following distal biceps repair using cortical buttons.

Methods: A retrospective review was conducted on all patients who underwent distal biceps repair with cortical buttons at a district general hospital between January 2014 and May 2022. Patient data, including age, gender, time from injury to surgery, type of procedure, and postoperative nerve injuries, were collected. The incidence of nerve injuries was analyzed, and the outcomes were assessed during postoperative follow-up visits.

Results: Ninety-six male patients were included in the study, with an average age of 45.6 years. The average time from injury to surgery was 22.6 days. All patients underwent primary repair except for two patients who underwent reconstruction with hamstring grafts. None of the patients experienced a PIN injury. However, 16 patients (16.7%) developed lateral cutaneous nerve injuries of the forearm, and three patients (3.1%) had superficial radial nerve injuries.

Conclusion: Our study, encompassing a large cohort of patients over an eight-year period, demonstrates the safety of distal biceps repair using cortical buttons with regard to PIN nerve injury. However, there were incidences of lateral cutaneous nerve of the forearm and superficial radial nerve injuries, consistent with previous studies.

## Introduction

Distal biceps tendon ruptures are relatively uncommon injuries, accounting for approximately 3% of all tendon ruptures in the upper limb [1]. They most commonly occur in the dominant arm of middle-aged and older individuals, with a peak incidence between 40 and 60 years of age [1]. Men are affected more frequently than women, with approximately 95% of all biceps tendon ruptures occurring in men [2]. The rupture of the distal biceps tendon typically occurs at its insertion point on the radial tuberosity, resulting from a forceful eccentric contraction of the biceps muscle while the elbow is in a flexed position [3-5]. This injury often leads to pain, weakness, and functional impairment in the affected arm.

Surgical repair for distal biceps tendon ruptures offers favorable outcomes in terms of pain relief, restoration of strength, and functional recovery [4,6]. The surgical techniques used for repair have evolved over the years, with the utilization of cortical buttons gaining popularity due to their biomechanical advantages and ability to provide stable fixation [7].

Despite the success of surgical treatment, concerns have been raised regarding potential complications associated with the procedure, particularly nerve injuries. The posterior interosseous nerve (PIN), lateral cutaneous nerve of the forearm, and superficial branch of the radial nerve are vulnerable to injury during the surgical approach and fixation of the distal biceps tendon [8].

Previous studies have reported variable rates of nerve injuries following distal biceps repair with the use of cortical buttons, with rates ranging around 5% for PIN injury and up to 36% for lateral cutaneous nerve of the forearm injury [7-12]. Although this is a relatively small percentage, the implications of this injury can be catastrophic for patients. As such, this increased risk of PIN injury has encouraged surgeons to avoid this operative technique in favor of newer repair methods. An example of this is the use of intramedullary buttons, which have been shown to have a poorer biomechanic profile when compared to cortical buttons [13]. Therefore, as per the current literature, there is a choice between optimal biomechanics and favorable complication profiles when deciding repair techniques.

The aim of this retrospective study was to determine the incidence of injury to the posterior interosseous nerve following distal biceps repair using cortical buttons. By evaluating a large number of patients, this study aimed to contribute valuable evidence regarding the safety and outcomes of this surgical technique in order to guide future practice.

## Materials and methods

This retrospective study was conducted through a review of patient records at a District General Hospital in the United Kingdom. All patients who underwent surgical repair using cortical buttons for distal biceps tendon ruptures between January 2014 and May 2022 were included in the study. Data were collected retrospectively, including patient demographics such as age and gender, as well as clinical variables such as side of injury, ASA grade, the time between injury and surgical intervention, and the type of procedure performed (primary repair versus reconstruction with grafts).

All patients included in this study underwent distal biceps repair with cortical buttons, and the surgical procedures were exclusively performed by experienced upper limb consultants. This ensured consistency in the surgical technique employed throughout the study. The Arthrex cortical button system was the consistent choice for all procedures, adding further uniformity to our analysis.

Within the scope of this study, we focused on assessing the incidence of specific nerve injuries of interest, namely the posterior interosseous nerve, superficial radial nerve, and lateral cutaneous nerve of the forearm. Our evaluation encompassed the entire postoperative follow-up period, during which patients attended outpatient clinic visits for comprehensive neurological assessments performed by experienced upper limb consultant surgeons. All patients were followed up for at least six months post-operatively, with data regarding their neurological assessment gathered during the early post-operative period (first 2-3 weeks).

The identification of an injury to the posterior interosseous nerve was based on documented evidence of a loss of finger or wrist extension, resulting in a finger or wrist drop. Similarly, an injury to the superficial radial nerve was determined by the presence of any loss of sensation on the dorsal and radial sides of the hand. On the other hand, the lateral cutaneous nerve of the forearm was assessed based on the occurrence of paraesthesia along the lateral forearm. We extracted this data from the clinical notes available in the hospital's electronic patient records system.

The collected data were analyzed, enabling us to determine the incidence of nerve injuries associated with the use of cortical buttons in distal biceps repair. By examining the results, we aimed to shed light on the safety and efficacy of this surgical approach, providing valuable insights for clinicians and researchers in the field.

Ethical approval was obtained from the institutional review board, ensuring that patient confidentiality and data protection regulations were followed throughout the study.

## Results

A total of 96 male patients were included in the study, with ages ranging from 21 to 71 years (mean age: 45.6 years). The time interval between the initial injury and surgical intervention had a mean duration of 22.6 days. The patients' American Society of Anaesthesiologists (ASA) grading is summarized in Table 1, indicating the overall health status of the patients involved in the study.

Regarding the type of procedure performed, the majority of patients (94 out of 96) underwent primary repair of the distal biceps tendon using cortical buttons. Two patients underwent reconstruction using hamstring grafts, which is a less common approach.

In terms of nerve injuries, none of the patients included in the study experienced injuries to the posterior interosseous nerve (PIN). However, 16 patients (16.7%) developed injury to the lateral cutaneous nerve of the forearm, and three patients (3.1%) experienced injury to the superficial radial nerve. These findings are summarized in Table 2, which presents the incidence of nerve injuries observed in the study population. 

**Table 1:** Distribution of Patients' ASA Grading

ASA Grade	Number of Patients
1	54
2	39
3	3

**Table 2:** Incidence of Nerve Injuries

Nerve Injured	Number of Patients
Posterior Interosseus Nerve (PIN)	0
Lateral Cutaneous Nerve	16
Superficial Radial Nerve	3

## Discussion

Distal biceps tendon repair is a well-established surgical intervention that aims to restore flexion and supination strength in patients with tendon ruptures. Surgical techniques for distal biceps repair have evolved over time, including the use of various fixation methods, such as suture anchors [14,15], cortical buttons [16,17], and intramedullary buttons [18]. These techniques have demonstrated favorable outcomes in terms of functional improvement and patient satisfaction [4].

Biomechanical studies have demonstrated higher load-to-failure rates of cortical buttons when compared to other techniques of distal biceps tendon repair [19]. Studies have also shown that this method allows for earlier post-operative rehabilitation as well as improved functional outcomes for patients [7,20]. However, the main disadvantage of the cortical button technique lies in its reportedly high rates of significant injury to the posterior interosseus nerve. This has led surgeons away from this technique in favor of newer techniques that lack the biomechanical advantage of cortical buttons but have a seemingly lower rate of complications.

Injury to the posterior interosseous nerve (PIN) is thought to be secondary to the placement of the button on the dorsal aspect of the cortex and the associated drilling that was needed [13]. Previous studies have reported varying rates of PIN injury following distal biceps repair, ranging from 1% to 5% [7-12]. These reports have raised concerns regarding the safety of using cortical buttons as a fixation method and highlighted the need for minimizing the risk of PIN injury.

In recent years, there has been a growing trend toward using intramedullary buttons for distal biceps repair, with the aim of reducing the risk of PIN injury [21]. This technique involves the placement of a button within the intramedullary canal of the radius, providing a stable and secure anchor point for tendon reattachment. This approach may have potential disadvantages, especially in cases where future surgical interventions, such as implant removal or treatment of infection, might be required.

In the present study, we aimed to assess the incidence of nerve injuries, particularly PIN injuries, associated with distal biceps repair using cortical buttons. This is the largest retrospective study of its nature, with a total of 96 patients investigated. Our results demonstrated a notable finding, as none of the patients included in the study experienced PIN injury over an eight-year period, encompassing a cohort of 96 patients. This finding supports the safety of using cortical buttons in relation to the PIN nerve and so provides with patients the benefit of having a more optimal fixation biomechanically without the risk of PIN nerve damage [19]. 

However, we did observe incidences of lateral cutaneous nerve of the forearm and superficial radial nerve injuries, which were comparable to rates reported in previous studies [7,9,11,12]. These injuries are likely attributed to traction or neuropraxia during the surgical approach rather than direct drilling through the bone. It is important to note that these nerve injuries generally have minimal functional implications and are often self-limiting. However, the occurrence of lateral cutaneous nerve injuries of the forearm and superficial radial nerve injuries warrants further attention and consideration during surgical planning and patient counseling.

Several limitations should be acknowledged in our study. Firstly, the study was conducted at a single center, which may limit the generalizability of the results. Secondly, the retrospective nature of the study introduces inherent biases and limitations in data collection. Additionally, there may be certain minor neurological complications that have been missed, including sensory disturbances not in keeping with injuries to the main three nerves being assessed in this study. However, a strength of our study lies in the large number of patients included and the consistent surgical approach performed by multiple upper limb consultants.

## Conclusions

Our study demonstrates the effectiveness and safety of cortical button repair for distal biceps tendon ruptures. The posterior interosseus nerve was preserved in all patients included in this study, while injury rates to the lateral cutaneous nerve and superficial radial nerve were comparable to other studies.

To further enhance our understanding of the safety and outcomes of distal biceps repair using cortical buttons, future prospective multicentric studies involving larger patient cohorts are warranted. These studies will provide more robust evidence and help determine the most optimal surgical techniques to minimize the risk of nerve injuries while achieving favorable functional outcomes in patients undergoing distal biceps repair.
